# Intelligent Sea States Identification Based on Maximum Likelihood Evidential Reasoning Rule

**DOI:** 10.3390/e22070770

**Published:** 2020-07-14

**Authors:** Xuelin Zhang, Xiaojian Xu, Xiaobin Xu, Diju Gao, Haibo Gao, Guodong Wang, Radu Grosu

**Affiliations:** 1 School of Automation, Hangzhou Dianzi University, Hangzhou 310018, Zhejiang, China; zhangxl0919@163.com (X.Z.); xxj03@hdu.edu.cn (X.X.); 2 Logistics Engineering College, Shanghai Maritime University, Shanghai 201306, China; djgao@shmtu.edu.cn; 3 School of Energy and Power Engineering, Wuhan University of Technology, Wuhan 430063, Hubei, China; hbgao_whut@126.com; 4 Institute of Computer Engineering, Vienna University of Technology, 1040 Vienna, Austria; guodong.wang@tuwien.ac.at (G.W.); radu.grosu@tuwien.ac.at (R.G.)

**Keywords:** sea states identification, propeller ventilation, MAKER rule, genetic algorithm

## Abstract

It is necessary to switch the control strategies for propulsion system frequently according to the changes of sea states in order to ensure the stability and safety of the navigation. Therefore, identifying the current sea state timely and effectively is of great significance to ensure ship safety. To this end, a reasoning model that is based on maximum likelihood evidential reasoning (MAKER) rule is developed to identify the propeller ventilation type, and the result is used as the basis for the sea states identification. Firstly, a data-driven MAKER model is constructed, which fully considers the interdependence between the input features. Secondly, the genetic algorithm (GA) is used to optimize the parameters of the MAKER model in order to improve the evaluation accuracy. Finally, a simulation is built to obtain experimental data to train the MAKER model, and the validity of the model is verified. The results show that the intelligent sea state identification model that is based on the MAKER rule can identify the propeller ventilation type more accurately, and finally realize intelligent identification of sea states.

## 1. Introduction

In recent years, marine electric propulsion technology has become more and more mature, and marine electric propulsion system has been widely used in ships with the rapid development of power electronic technology [1,2]. During ship navigation, the stability of propulsion system and the ship security are often affected by the continuous changes of sea states. When a ship is traveling in the normal sea state, the external environment has little effect on its navigation. In this ideal state, the ship can work stably and the propeller will not produce propeller ventilation. However, in the extreme sea state, the external environment will have great impact on ship navigation (e.g., thrust loss, vibration, turbulence, etc.) and lead to the propeller ventilation, and even cause the failure of various mechanical equipment in the propulsion system [3,4]. To ensure the stability and safety of the navigation, it is necessary to adopt the appropriate control strategy according to the sea state. In the normal sea state, the propeller is fully submerged in water. There is no air circulation between the blades, and the propeller will not produce propeller ventilation. In this occasion, the speed control strategy is adopted in order to realize the control of propulsion system [5]. However, the navigation and operation environment of ship will gradually evolve into extreme sea conditions with the continuous navigating to the deep sea. When a ship sails under extreme sea conditions, the propeller will enter and exit the sea surface frequently, and the propeller will experience a large instantaneous load change. Eventually, it will cause ventilation on the propeller. To better control the propulsion system, Sorensen et al. [6] proposed an anti-spin control strategy, which can increase the propeller thrust until the end of the ventilation. Therefore, it is significant to identify the sea state and adopt the reasonable control strategy, so that the propulsion system can be well controlled in any sea state.

When considering the importance of sea states identification, experts and scholars have been working on finding simpler and more effective identification methods to meet the demands of complex sea conditions. So far, most of the existing ventilation identification methods have been designed based on the dynamic modeling of water and the propeller. In general, these methods only require information or accurate estimates of vessel specific parameters. In reference [7], Califano and Steen proposed that the degree and type of ventilation should depend on the propeller advance ratio and immersion ratio and a series of numerical simulation experiments were carried out on the ventilation to clarify the influence of the ventilation on the propeller. Smogeli et al. [8,9,10,11] conducted a series of simulation experiments and control strategy researches on the ventilation generated by the propeller under extreme sea conditions and proposed a propeller torque loss estimation method that was based on empirical formulas to realize the identification of propeller ventilation and then determine the type of sea state. This method of ventilation identification based on empirical formulas has realized the effective identification of ventilation to some certain extent, but the method has a weak migration ability and it has great limitations in practical applications. Kozlowska et al. [12] proposed a method to recognize the ventilation type based on fuzzy logic inference, which categorized different ventilation types and inception mechanisms. However, this method only considers full ventilation without further studying the severity of ventilation. In reference [13], Savio et al. proposed a method based on fuzzy logic inference to identify the sea state. However, this method involves a lot of manual operations, and it is not suitable for the case in which a large amount of continuous monitoring data is provided.

Ventilation identification belongs to the field of classification problems. Machine learning has powerful data processing capabilities and can solve classification problems effectively within limited conditions. There are various solutions to solve this problem (e.g., classifier based on genetic algorithm, classifier based on back propagating artificial neutral net (BP-ANN), classifier based on support vector machine (SVM), etc.). Genetic algorithm [14,15] is a method to search for the optimal solution by simulating natural evolution process. It has a wide range of applications and scalability, and it is easy to be combined with other algorithms. BP-ANN [16] has strong nonlinear mapping ability, which can approximate any nonlinear continuous functions with any precision, and it also has high self-learning and self-adaptive ability. SVM is a common machine learning method in the field of classification. If the data are complete and the sample size is small, SVM can effectively solve the classification problem. Machine learning methods can effectively solve classification problems to a certain extent, but they all have certain limitations. When considering the problems of these methods (e.g., poor portability, low precision, and strong subjectivity, etc.), we try to find a modeling method that can comprehensively amplify the advantages of the methods mentioned above and avoid their disadvantages. Yang and Xu [17] proposed the maximum likelihood evidential reasoning (MAKER) framework, which can solve the problems in ventilation identification methods. As described in reference [17], the MAKER framework is a process of making data-driven inferences from inputs to outputs, under uncertainty. The state space model (SSM) and evidence space model (ESM) are fully considered in MAKER framework, and the fusion rules between independent evidence are illustrated as MAKER rule [17,18]. The MAKER rule can generate specific inference rules (e.g., evidential reasoning (ER) rule, Dempster rule and Bayes rule) under various conditions, which has four advantages: (1) on the evidence fusion and inference based on MAKER rule is completely a data-driven method, and therefore it does not need to make any assumptions on the relationships between input variables and output parameters; (2) in the MAKER rule, the interdependence between each piece of evidence is fully considered and the method based on MAKER rule can realize the parameter estimation by using incomplete data samples; (3) being different from Bayes rule, the MAKER framework does not rely on a priori probabilities; and, (4) compared with some “black box” methods (e.g., back propagation neural network (BP), etc.), the physical meaning of the parameters in MAKER is clear, readily understood, and adjustable.

This paper further refines the ventilation degree and divides it into non-ventilation region, partial ventilation region, and full ventilation region based on the ventilation effects on the propeller when it is in and out of sea [7]. Specifically, the sea state when the propeller is in the non-ventilation region is identified as the normal sea state, and the sea state when the propeller is in the partial or full ventilation region is identified as the extreme sea state (that is, the sea state is normal when the propeller does not produce the ventilation; otherwise, the sea state is extreme). This paper proposes an intelligent identification method for sea states that is based on the MAKER rule in order to effectively identify the current sea state. Firstly, the initial reference evidence matrix (IREM) and the joint reference evidence matrix (JREM) are constructed based on the sample casting and the normalization of likelihood function. Secondly, different pieces of evidence are fused and inferenced based on the MAKER rule to identify the ventilation. Subsequently, this paper will identify the sea state based on the correspondence between the type of propeller ventilation and sea state. Finally, GA is used to optimize the parameters of propeller ventilation model based on MAKER rule, and the validity of the model is verified based on the experiment data that are generated by MATLAB/SIMULINK simulation.

The main contributions of this work are as follows: (1) in this paper, an intelligent sea states identification model based on MAKER rule is established, which can identify the ventilation of propeller and the sea state more accurately. (2) In the model of sea states identification based on the MAKER rule, the independence between the evidence obtained from different input variables is fully considered, compensating the shortage of ER rule that can only combine independent evidence. (3) In the process of evidence fusion using the MAKER rule, this paper proposes a method for obtaining the reliability factor of evidence based on the basic probability mass function.

The rest of the article is arranged, as follows. Section 2 briefly introduces the propeller ventilation and MAKER rule. Section 3 focuses on the modeling and reasoning process of intelligent sea states identification model that is based on the MAKER rule. In Section 4, the newly proposed model is applied to identify sea states during a ship’s navigation that is simulated in SIMULINK, and the performance of the model is compared with that of the existing methods. Section 5 summarizes the work conducted in the article and the advantages of the MAKER model.

## 2. Preliminaries

### 2.1. Propeller Ventilation

When a ship is navigating, the propeller will be affected by the external environment, and it will enter and exit the surface of the sea. In the normal sea state, the propeller is fully submerged in water and there is no air circulation between blades. However, the propeller will be exposed to the surface or near the surface with the change of the sea environment in the extreme sea state. In this condition, the loss of thrust and torque will be caused by the increase of the vertical motion amplitude of propeller [19,20,21]. When the propeller has a high load and has been exposed or close to the water surface, the low pressure on its blades will form an air funnel, which makes it suck the air on the water surface, and the airflow between the blades causes the propeller to lose its grip [22]. At the same time, the rotating water flow generated by the propeller will distort the water surface, and eventually form a vortex near the propeller, so that the propeller blades are covered with air. This phenomenon is called propeller ventilation.

**Figure 1:**
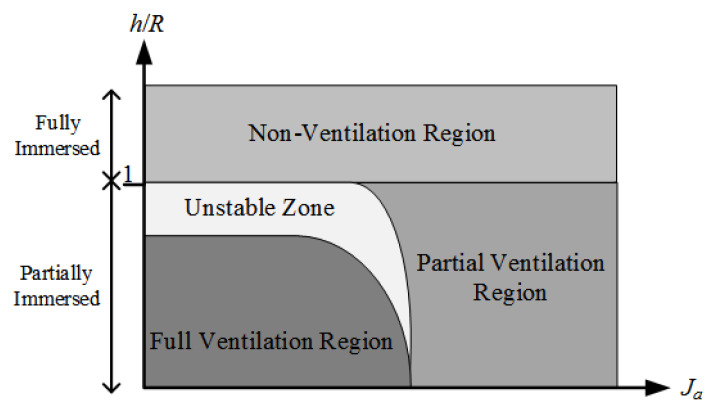
Ventilation regions.

Propeller advance ratio $J_{a}$ and immersion ratio h/R (ratio of propeller immersion *h* to radius *R*) are used as two important indicators to measure the degree of propeller ventilation [7,8]. The ventilation can be divided into non-ventilation region, partial ventilation region, and full ventilation region according to the degrees [7,8], as shown in Figure 1. The measurement criteria for specific regions are as follows.

- Non-ventilation region: propeller is immersed in deep water or the load is low when the water is shallow.
- Partial ventilation region: propeller produces ventilation, but it does not act on the entire propeller. In other words, the degree of the ventilation and the position of the air cavity on the propeller change with time. When the propeller has a high advance ratio, this state can exist for a long time. Otherwise, this region is unstable with a small advance ratio. The actual operating condition of the propeller varies between non-convolution region and full ventilation region.
- Full ventilation region: a single air cavity covers each blade of the propeller, which means that the pressure of the propeller is almost equal to atmospheric pressure, which is a relatively stable state.

### 2.2. MAKER Rule

Suppose that $\Theta =\{h_{1},h_{2},\ldots ,h_{N}\}$ is a frame of discernment, which contains *N* propositions that are mutually exclusive and collectively exclusive. Θ and all of its subsets constitute the power set, denoted by P(Θ) or $2^{\Theta }$. The basic probability distribution of system input in MAKER framework is shown in Equation (1):

(1) $$e_{i,j}=\left\{\left(e_{i,j}\left(\theta \right),p_{\theta ,i,j}\right)|\forall \theta \subseteq \Theta \mathrm{and}\sum_{\theta \subseteq \Theta }p_{\theta ,i,j}=1\right\}$$

where $e_{i,j}$ denotes the ith piece of evidence from the jth input variable $x_{j}$ and $e_{i,j}\left(\theta \right)$ denotes the element of evidence $e_{i,j}$, pointing to proposition θ. $p_{\theta ,i,j}$ denotes the degree of $e_{i,j}$ supporting the proposition θ.

In MAKER rule, the reliability factor and importance weight of $e_{i,j}$ are denoted by $r_{i,j}$ and $w_{i,j}$ respectively. The reliability factor $r_{i,j}$ of $e_{i,j}$ is as follows.

(2) $$r_{i,j}=\sum_{\theta \in \Theta }r_{\theta ,i,j}p\left(e_{i,j}\left(\theta \right)\right)$$

where $p(e_{i,j}\left(\theta \right))$ denotes the degree of $e_{i,j}$ supporting the proposition θ. $r_{\theta ,i,j}=p\left(\theta |e_{i,j}\left(\theta \right)\right)$ represents the reliability of evidential element $e_{i,j}\left(\theta \right)$. In this sense, it is defined as the conditional probability that proposition θ is true when $e_{i,j}$ points to proposition θ in reference [17]. $r_{\theta ,i,j}$ essentially measures the quality of $e_{i,j}$, which considers how data are generated and how $e_{i,j}$ can be acquired from data.

If $e_{i,j}$ and other evidence are obtained from the same data source, then the probability mass of evidence $e_{i,j}$ supporting proposition θ is

(3) $$m_{\theta ,i,j}=r_{\theta ,i,j}p\left(e_{i,j}\left(\theta \right)\right)$$

If $e_{i,j}$ and other evidence are obtained from different data sources, then the probability mass of evidence $e_{i,j}$ supporting proposition θ is defined as in Equation (4):

(4) $$m_{\theta ,i,j}=w_{\theta ,i,j}p_{l}\left(e_{i,j}\left(\theta \right)\right)$$

where $p_{l}$ represents the probability function and $p_{l}\left(e_{i,j}\left(\theta \right)\right)$ represents the basic probability that $e_{i,j}$ points to θ. $w_{\theta ,i,j}=w_{i,j}p_{l}\left(\theta |e_{i,j}\left(\theta \right)\right)$ represents the importance weight of $e_{i,j}\left(\theta \right)$, and $w_{i,j}$ is a non-negative constant. When $p=p_{l}$, $w_{\theta ,i,j}=r_{\theta ,i,j}$, which means $w_{i,j}$ = 1.

Let $e_{i,j}$ and $e_{l,m}$ denote the ith piece of evidence from the input variable $x_{j}$ and the lth piece of evidence from the input variable $x_{m}$, respectively. For two pieces of evidence $e_{i,j}$ and $e_{l,m}$ with interdependence, the joint support degree $p_{\theta ,e(2)}$ is defined as in Equation (5):

(5) $$\begin{array}{c} \;\;p_{\theta ,e(2)}=\left\{\begin{array}{ccc} 0 & & \theta =\phi \\ \frac{{\tilde{m}}_{\theta ,e(2)}}{\sum_{D\subseteq \Theta }{\tilde{m}}_{D,e(2)}} & & \theta \subseteq \Theta ,\theta \neq \phi \end{array}\right.\\ {\tilde{m}}_{\theta ,e(2)}=\left[\left(1-r_{l,m}\right)m_{\theta ,i,j}+\left(1-r_{i,j}\right)m_{\theta ,l,m}\right]\\ \;\;\;\;\;\;\;\;\;\;\;\;+\sum_{B\cap C=\theta }\gamma_{B,C,ij,lm}\mu_{B,C,ij,lm}m_{B,i,j}m_{C,l,m}\;\;\;\;\forall \theta \subseteq \Theta \end{array}$$

where γB,C,ij,lm=rij,lmrij,lm(ri,jrl,m)(ri,jrl,m) is a non-negative coefficient, which represents the ratio between the joint reliability of evidence $e_{i,j}$ and $e_{l,m}$ and the product of their individual reliabilities. It reflects the degree of joint support for θ from both $e_{i,j}$ and $e_{l,m}$ relative to their individual support given that $e_{i,j}$ points to proposition *B* and $e_{l,m}$ points to proposition *C*. $\mu_{B,C,ij,lm}$ represents the degree of interdependence between the evidential elements $e_{i,j}\left(B\right)$ and $e_{i,j}\left(C\right)$, denoted by “interdependence index”, which is defined, as follows

(6) μB,C,ij,lm=0if pB,i,j=0orpC,l,m=0pB,C,ij,lmpB,C,ij,lm(pB,i,jpC,l,m)(pB,i,jpC,l,m)otherwise

When fusing *K* pieces of evidence $e_{k}$ (*k* = 1,2,…,*K*) with interdependence, the recursion formula of MAKER rule that is given in Equation (7) can be used to integrate multiple pieces of evidence.

(7) $$\begin{array}{c} p_{\theta ,e(K)}=\left\{\begin{array}{ccc} 0 & & \theta =\phi \\ \frac{{\tilde{m}}_{\theta ,e(K)}}{\sum_{D\subseteq \Theta }{\tilde{m}}_{D,e(K)}} & & \theta \subseteq \Theta ,\theta \neq \phi \end{array}\right.\\ {\tilde{m}}_{\theta ,e(k)}=\left[\left(1-r_{k}\right)m_{\theta ,e(k-1)}+m_{P(\Theta ),e(k-1)}m_{\theta ,k}\right]\\ \;\;\;\;\;\;\;\;\;\;\;\;+\sum_{B\cap C=\theta }\gamma_{B,C,e_{1},\ldots ,e_{k}}\mu_{B,C,e_{1},\ldots ,e_{k}}m_{B,e(k-1)}m_{C,i}\;\;\;\forall \theta \subseteq \Theta \\ {\tilde{m}}_{P(\Theta ),e(k)}=\left(1-r_{k}\right)m_{P(\Theta ),e(k-1)} \end{array}$$

It should be noted that the above MAKER rule can generate specific inference rules under various conditions. If $w_{\theta ,i,j}=w_{i,j}$ for any B,C,θ⊆Θ, then the MAKER rule can be reduced to the evidential reasoning (ER) rule [23]. If $r_{i,j}=w_{i,j}=1$ is further assumed, then the MAKER rule can be reduced to Dempster’s rule [24].

## 3. Intelligent Sea States Identification Based on MAKER Rule

### 3.1. Framework of Sea States Identification

**Figure 2:**
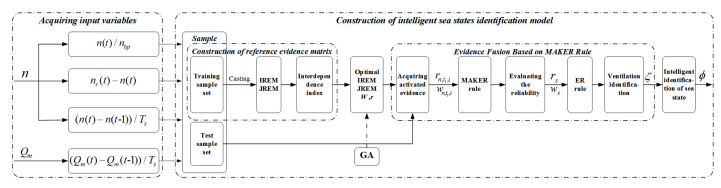
Framework of maximum likelihood evidential reasoning (MAKER) rule-based sea state identification.

When the ship sails under different sea conditions, the degree of ventilation that is generated by the propeller is different. In this paper, a sea states identification model is developed based on the MAKER rule to distinguish different ventilation types and identify the sea state according to the correspondence between the type of propeller ventilation and sea states. The framework of the sea states identification model that is based on the MAKER rule is shown in Figure 2.

The intelligent identification of sea states based on MAKER rule can be implemented by three steps:

1. Acquiring input variables.
  In ship navigation, there may be many problems in data acquisition. This paper uses the measurable data of the propulsion system to construct the model, including the actual speed *n* and actual torque $Q_{m}$ of the propulsion motor. To realize the intelligent sea states identification more effectively, this paper converts the sampled parameters $Q_{m}$ and *n* into parameters that can describe the dynamic change performance of the system (i.e., relative speed $n\left(t\right)/n_{bp}$ representing the current operating conditions, speed following performance indicator $n_{r}\left(t\right)-n\left(t\right)$, speed fluctuation performance indicator $\left(n\left(t\right)-n\left(t-1\right)\right)/T_{s}$, torque fluctuation performance indicator $\left(Q_{m}\left(t\right)-Q_{m}\left(t-1\right)\right)/T_{s})$, and the above four parameters are selected as the input variables of the system, where $n_{r}\left(t\right)$ represents the target speed of the propeller, $T_{s}$ represents the sampling period, and $n_{bp}$ represents the maximum speed of the propeller.
2. Construction of intelligent sea states identification model.
  The intelligent sea states identification model that is based on the MAKER rule can be divided into three steps. Specifically, the ventilation identification model based on the MAKER rule is constructed through steps (a) and (b) to realize the identification of ventilation, and in step (c), the sea states can be evaluated based on the identifying result of ventilation. The three steps is described in detail, as follows:
  (a) Construction of reference evidence matrix.
    The initial reference evidence matrix (IREM) and the joint reference evidence matrix (JREM) are constructed based on the sample set, and the interdependence index $\mu_{n,t_{1},\ldots ,t_{i},\ldots ,t_{M}}^{1,\ldots ,i,\ldots ,M}$ between reference evidence is generated according to JREM and IREM. The reasoning process of REM and the interdependence index is detailed in steps A to B in Section 3.2.1.
  (b) Evidence fusion based on MAKER rule.
    The MAKER rule is used to fuse the referential evidence sets (e.g., $\left\{e_{t_{1}}^{1},\ldots ,e_{t_{i}}^{i},\ldots ,e_{t_{M}}^{M}\right\}$, $\left\{e_{t_{1}}^{1},\ldots ,e_{t_{i}}^{i},\ldots ,e_{t_{M}+1}^{M}\right\}$, etc.) activated by the input vector $X\left(k\right)=[f_{1}\left(k\right),\ldots ,f_{i}\left(k\right),\ldots ,f_{M}\left(k\right)]$ to generate the activated evidence $e_{s}$. Subsequently, the ER rule is used to fuse $e_{1},\ldots ,e_{s},\ldots ,e_{2^{M}}$, and the ventilation type is identified according to the fusing result. The reasoning process is detailed in steps A to C in Section 3.2.2.
  (c) Intelligent identification of sea states.
    The recognition result of the ventilation type can be obtained from the reasoning process of steps (a) and (b). According to the correspondence between the type of ventilation and sea states, the type of sea state can be judged according to the type of the ventilation.
3. Optimization of the ventilation identification model based on GA.
  The MAKER model constructed by the initial parameters may not accurately capture the complex nonlinear relationship between the input feature $f_{i}\left(i=1,2,\ldots ,M\right)$ and the output $F_{n}$. Therefore, the minimum mean square error (MSE) between the estimated probability (${\hat{p}}_{n,k}$) and the real probability ($p_{n,k}$) of the ventilation type is selected as the objective function, and GA is used to optimize the parameters of ventilation identification model to improve the accuracy of ventilation identification, and finally realize the intelligent identification of sea states.

### 3.2. Construction of Intelligent Sea States Identification Model

The intelligent sea states identification model based on MAKER rule includes three parts: (a) construction of reference evidence matrix; (b) evidence fusion based on MAKER rule; and, (c) intelligent identification of sea states. The detailed modeling and inference process are described detailedly in the following.

#### 3.2.1. Construction of Reference Evidence Matrix

#### A. Generate Evidence from Data Samples

**Step 1:**$X\left(k\right)=[f_{1}\left(k\right),\ldots ,f_{i}\left(k\right),\ldots ,f_{M}\left(k\right)]$ is the input of the sea states identification model based on MAKER rule. The reference value set of input variable $f_{i}$ is $A_{i}=\left\{A_{1}^{i},\ldots ,A_{t_{i}}^{i},\ldots ,A_{T_{i}}^{i}|t_{i}=1,2,\ldots ,T_{i}\right\}$, where *M* is the number of input variables, and $T_{i}$ is the number of reference values for $f_{i}$. $\Theta =\{F_{n}\;|n=1,2,\ldots ,N\}$ represents the set of propeller ventilation types, where $F_{n}$ represents the *n*th ventilation type in Θ, and *N* is the number of ventilation types. Firstly, transform the relationship between the input variable $f_{i}$ and the output type $F_{n}$ into the relationship between the reference value set $A_{i}$ for $f_{i}$ and the output type $F_{n}$. Note that the reference value of input $A_{i}$ can be adjusted, and the initial value can be given by domain experts or given randomly, and then optimized by using training samples. Subsequently, the similarity distribution of the specific input $X\left(k\right)=f_{i}\left(k\right)$ to its reference values $A_{i}$ can be expressed in belief distribution as Equation (8) [25].

(8) $$S_{P}\left(f_{i}\left(k\right)\right)=\left\{\left(A_{t_{i}}^{i},\alpha_{i,t_{i}}\right)|t_{i}=1,2,\ldots ,T_{i};i=1,2,\ldots ,M\right\}$$

where $\alpha_{i,t_{i}}$ indicates the similarity between $f_{i}\left(k\right)$ and the reference value $A_{t_{i}}^{i}$, and $\alpha_{i,t_{i}}$ can be calculated according to Equation (9).

(9) $$\left\{\begin{array}{cc} \alpha_{i,t_{i}}=\frac{A_{t_{i}+1}^{i}-f_{i}\left(k\right)}{A_{t_{i}+1}^{i}-A_{t}^{i}};\alpha_{i,t_{i}+1}=\frac{f_{i}\left(k\right)-A_{t_{i}}^{i}}{A_{t_{i}+1}^{i}-A_{t_{i}}^{i}} & A_{t_{i}}^{i}\leq f_{i}\left(k\right)\leq A_{t_{i}+1}^{i}\\ \alpha_{i,1}=1;\alpha_{i,t}=0\left(t_{i}\neq 1\right) & f_{i}\left(k\right)\leq A_{1}^{i}\\ \alpha_{i,T_{i}}=1;\alpha_{i,t}=0\left(t_{i}\neq T_{i}\right) & f_{i}\left(k\right)\geq A_{T_{i}}^{i} \end{array}\right.$$

**Step 2:** all of the sample pairs in data set $S_{Train}$ are represented by integrated similarity which can be used to generate the casting result reflecting the relationship between the input reference values and the output type, as shown in Table 1.

where $a_{n,t_{i}}$ represents the sum of the integrated similarity of $f_{i}\left(k\right)$ in all sample pairs $\left(f_{i}\left(k\right),F_{n}\left(k\right)\right)$ matching the reference value $A_{t_{i}}^{i}$ while the output type is $F_{n}$. $\eta_{t_{i}}=\sum_{n=1}^{N}a_{n,t_{i}}$ and $\delta_{n}=\sum_{t_{i}=1}^{T_{i}}a_{n,t_{i}}$ represent the sum of the integrated similarity of all $f_{i}\left(k\right)$ matches $A_{t_{i}}^{i}$ and the output of the samples in $S_{Train}$ matching $F_{n}$, respectively, which satisfy $\sum_{n=1}^{N}\delta_{n}=\sum_{t_{i}=1}^{T_{i}}\eta_{t_{i}}=K$. *K* represents the total number of samples in $S_{Train}$, satisfying *k* = 1,2,…,*K*.

According to Table 1, the likelihood function $c_{n,t_{i}}$, which indicates that $f_{i}\left(k\right)$ equals $A_{t_{i}}^{i}$ and output type matches $F_{n}$ can be acquired by Equation (10).

(10) $$c_{n,t_{i}}=p\left(A_{t_{i}}^{i}|F_{n}\right)=\frac{a_{n,t_{i}}}{\delta_{n}}$$

The IREM that is shown in Table 2 describes the mapping relationship between the input $f_{i}$ and the output type $F_{n}$. In Table 2, $e_{t_{i}}^{i}=[\beta_{1,t_{i}}^{i},\ldots ,\beta_{n,t_{i}}^{i},\ldots ,\beta_{N,t_{i}}^{i}]$ represents the evidence corresponds to the reference value $A_{t_{i}}^{i}$ for input $f_{i}$, where $\beta_{n,t_{i}}^{i}$ represents the belief degree that evidence $e_{t_{i}}^{i}$ supporting $F_{n}$, satisfying $\sum_{n=1}^{N}\beta_{n,t_{i}}^{i}=1$. $\beta_{n,t_{i}}^{i}$ can be calculated by normalizing the likelihood function values $c_{n,t_{i}}$ according to Equation (11).

(11) $$\beta_{n,t_{i}}^{i}=\frac{c_{n,t_{i}}}{\sum_{k=1}^{N}c_{k,t_{i}}}$$

#### B. Interdependence between Pairs of Evidence

When fusing multiple pieces of reference evidence obtained from different inputs, the interdependence index is introduced to describe the interdependence between pairs of evidence. This section specifically introduces the calculation of the interdependence index, as shown in Equation (6). According to Equation (6), the interdependence between multiple pieces of reference evidence can be calculated according to Equation (12).

(12) $$\mu_{n,t_{1},\ldots ,t_{i},\ldots ,t_{M}}^{1,\ldots ,i,\ldots ,M}=\left\{\begin{array}{ccc} 0 & & if\beta_{n,t_{1}}^{1}\beta_{n,t_{2}}^{2}\cdots \beta_{n,t_{M}}^{M}=0\\ \frac{\beta_{n,t_{1},\ldots ,t_{i},\ldots ,T_{M}}^{1,\ldots ,i,\ldots ,M}}{\beta_{n,t_{1}}^{1}\beta_{n,t_{2}}^{2}\cdots \beta_{n,t_{M}}^{M}} & & otherwise \end{array}\right.$$

where $\beta_{n,t_{1},\ldots ,t_{i},\ldots ,t_{M}}^{1,\ldots ,i,\ldots ,M}$ denotes the joint belief degree of $e_{t_{1},\ldots ,t_{i},\ldots ,t_{M}}^{1,\ldots ,i,\ldots ,M}$ supporting the proposition $F_{n}$. $e_{t_{1},\ldots ,t_{i},\ldots ,t_{M}}^{1,\ldots ,i,\ldots ,M}$ is the combination of referential evidences. The joint belief degree is generated, as follows:

If multiple input variables are taken into consideration simultaneously, the joint similarity distribution of the specific input vector $X\left(k\right)=[f_{1}\left(k\right),\ldots ,f_{i}\left(k\right),\ldots ,f_{M}\left(k\right)]$ can be transformed into the belief distribution for the combination of referential values $A_{t_{1},\ldots ,t_{i},\ldots ,t_{M}}^{1,\ldots ,i,\ldots ,M}$.

(13) $$S_{I}\left(\left[f_{1}\left(k\right),\ldots ,f_{M}\left(k\right)\right]\right)=\left\{\left(A_{t_{1},\ldots ,t_{i},\ldots ,t_{M}}^{1,\ldots ,i,\ldots ,M},\alpha_{1,\ldots ,i,\ldots ,M,t_{1},\ldots ,t_{i},\ldots ,t_{M}}\right)|t_{i}=1,2,\ldots ,T_{i};i=1,2,\ldots ,M\right\}$$

where $A_{t_{1},\ldots ,t_{i},\ldots ,t_{M}}^{1,\ldots ,i,\ldots ,M}=\left\{A_{t_{1}}^{1},\ldots ,A_{t_{i}}^{i},\ldots ,A_{t_{M}}^{M}\right\}$ and $\alpha_{1,\ldots ,i,\ldots ,M,t_{1},\ldots ,t_{i},\ldots ,t_{M}}=\alpha_{1,t_{1}}*\cdots *\alpha_{i,t_{i}}*\cdots *\alpha_{M,t_{M}}$.

In Equation (13), $\alpha_{1,\ldots ,i,\ldots ,M,t_{1},\ldots ,t_{i},\ldots ,t_{M}}$ represents the joint similarity between $\left[f_{1}\left(k\right),\ldots ,f_{i}\left(k\right),\ldots ,f_{M}\left(k\right)\right]$ and the combination of referential values $A_{t_{1},\ldots ,t_{i},\ldots ,t_{M}}^{1,\ldots ,i,\ldots ,M}$, and $\alpha_{i,t_{i}}$ can be calculated by the piecewise linear function as Equation (9).

Being consistent with the evidence acquisition method described in step A in Section 3.2.1, all of the sample pairs in data set $S_{Train}$ are represented by integrated similarity which can be used to generate the casting result, reflecting the relationship between the combination of referential values and the output type. Subsequently, the joint belief degree $\beta_{n,t_{1},\ldots ,t_{i},\ldots ,t_{M}}^{1,\ldots ,i,\ldots ,M}$ of $e_{t_{1},\ldots ,t_{i},\ldots ,t_{M}}^{1,\ldots ,i,\ldots ,M}$ supporting the proposition $F_{n}$ can be calculated by normalizing the joint likelihood function values $c_{n,t_{1},t_{2}}$. The following will illustrate the reasoning process of joint belief degree $\beta_{n,t_{1},t_{2}}^{1,2}$ when the input vector is $X\left(k\right)=[f_{1}\left(k\right),f_{2}\left(k\right)]$. At the same time, the joint casting result of input vector $X\left(k\right)=[f_{1}\left(k\right),f_{2}\left(k\right)]$ is given in Table 3.

According to Table 3, the joint likelihood function $c_{n,t_{1},t_{2}}$ indicating that the combination of referential values equals to $A_{t_{1},t_{2}}^{1,2}$ (i.e., [$A_{t_{1}}^{1}$,$A_{t_{2}}^{2}$]) and output type matches can be acquired by Equation (14).

(14) $$c_{n,t_{1},t_{2}}=p\left(A_{t_{1},t_{2}}^{1,2}|F_{n}\right)=\frac{a_{t_{1},t_{2}}^{n}}{\delta_{n}}$$

The JREM that is shown in Table 4 describes the mapping relationship between the input vector [$f_{1}$,$f_{2}$] and the output type $F_{n}$. In Table 4, $e_{n,t_{1},t_{2}}^{1,2}=[\beta_{1,t_{1},t_{2}}^{1,2},\ldots ,\beta_{n,t_{1},t_{2}}^{1,2},\ldots ,\beta_{N,t_{1},t_{2}}^{1,2}]$ represents the evidence that the input vector [$f_{1}$,$f_{2}$] corresponds to the combination of referential values $A_{t_{1},t_{2}}^{1,2}$ (i.e., $f_{1}$ matching $A_{t_{1}}^{1}$ and $f_{2}$ matching $A_{t_{2}}^{2}$), where $\beta_{n,t_{1},t_{2}}^{1,2}$ represents the joint belief degree of output type matching $F_{n}$ in the evidence $\beta_{n,t_{1},t_{2}}^{1,2}$, satisfying $\sum_{n=1}^{N}\beta_{n,t_{1},t_{2}}^{1,2}=1$. $\beta_{n,t_{1},t_{2}}^{1,2}$ can be calculated by normalizing the likelihood function values according to Equation (15).

(15) $$\beta_{n,t_{1},t_{2}}^{1,2}=\frac{c_{n,t_{1},t_{2}}}{\sum_{k=1}^{N}c_{k,t_{1},t_{2}}}$$

The joint belief degree of input vector $X\left(k\right)=[f_{1}\left(k\right),f_{2}\left(k\right)]$ can be calculated by the above process, and the interdependence index between the combination of reference evidence $e_{t_{1}}^{1}$ and $e_{t_{2}}^{2}$ can be obtained by Equation (12). When the input vector is $X\left(k\right)=[f_{1}\left(k\right),\ldots ,f_{i}\left(k\right),\ldots ,f_{M}\left(k\right)]$, the joint belief degree can be acquired using the above reasoning process as well.

#### 3.2.2. Evidence Fusion Based on MAKER Rule

#### A. Acquiring Activated Evidence

**Figure 3:**
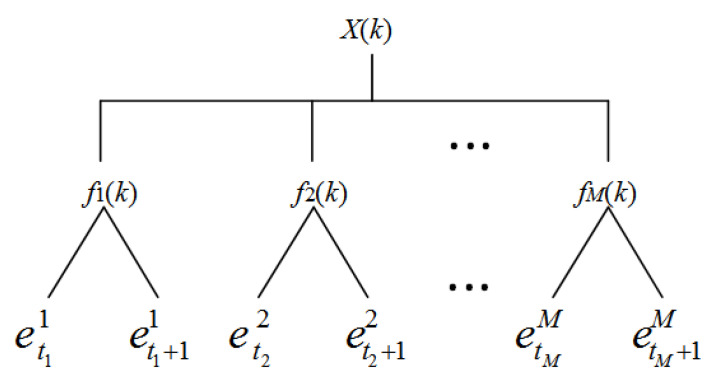
Evidence activated by input variables.

**Figure 4:**
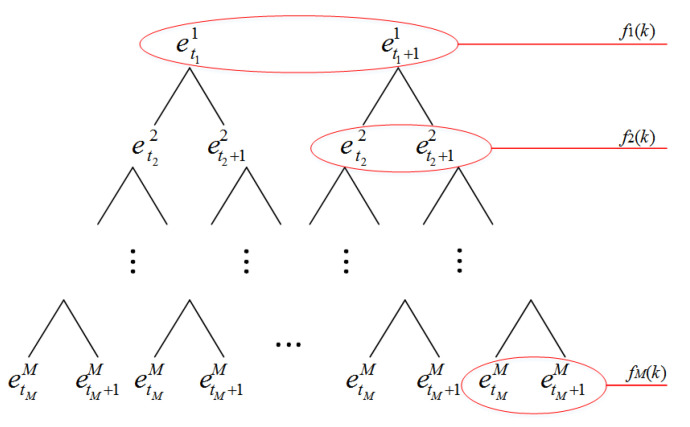
Activated reference evidence combination.

The input variable $f_{i}\left(k\right)$ is obtained from specific input vector $X\left(k\right)=[f_{1}\left(k\right),\ldots ,f_{i}\left(k\right),\ldots ,f_{M}\left(k\right)]$, which necessarily satisfies $A_{t_{i}}^{i}<f_{i}\left(k\right)<A_{t_{i}+1}^{i}$. In this condition, $f_{i}\left(k\right)$ activates two adjacent pieces of evidence $e_{t_{i}}^{i}$ and $e_{t_{i}+1}^{i}$ corresponding to reference values $A_{t_{i}}^{i}$ and $A_{t_{i}+1}^{i}$. Consequently, 2*M* pieces of reference evidence (Figure 3) are activated, generating $2^{M}$ combined reference evidences, and each combination of evidence includes *M* pieces of reference evidence. The structure of the binary tree is used to show the combination of evidences, as shown in Figure 4. The node of the *i*th layer in the binary tree represents the activated reference evidence for the input variable $f_{i}\left(k\right)$. The combination of the nodes on each path of the binary tree is the combination of reference evidence.

At this time, the 2*M* pieces of combined evidence are activated by input vector X(k) (e.g., $\left\{e_{t_{1}}^{1},\ldots ,e_{t_{i}}^{i},\ldots ,e_{t_{M}}^{M}\right\}$, $\left\{e_{t_{1}}^{1},\ldots ,e_{t_{i}}^{i},\ldots ,e_{t_{M}+1}^{M}\right\}$, etc.). The MAKER rule presented in Equation (7) is used to fuse the $2^{M}$ pieces of combined evidence, and the result can be expressed as in Equation (16).

(16) $$e_{s}=\left\{\left(F_{n},p_{n,e(M)}\right),s=1,\ldots ,2^{M}\right\}$$

Because the importance weight and reliability of the evidential elements $e_{t_{i}}^{i}\left(F_{n}\right)$ can be adjusted, the initial value of $w_{n,t_{i},i}$ and $r_{n,t_{i},i}$ can be considered as 1, i.e., $w_{n,t_{i},i}=r_{n,t_{i},i}=1$. The reliability of the reference evidence can be obtained by Equations (8) and (9), and $w_{t_{i},i}=r_{t_{i},i}$. The reliability ratio $\gamma_{B,C,e_{t_{1}}^{1},\ldots ,e_{t_{M}}^{M}}$ is also an adjustable parameter, with an initial value of 1.

#### B. Evaluating the Reliability of Evidence

Before fusing evidence, the reliability and importance weight of the evidence $e_{s}$ need to be obtained. In general, the reliability of the evidence has a positive correlation with the importance weight, so we can assume that they are equal with each other. To obtain the reliability of evidence, this paper proposes a method to obtain the reliability factor of evidence based on the basic probability mass function. According to the definition of basic probability mass function in reference [23], it can be proved that the reliability of the evidence $e_{s}$ is $r_{s}=\sum_{n\subseteq \Theta }m_{n,s}=1-m_{P(\Theta ),s}$ when the importance weight of evidence is equal to the reliability of the evidence (i.e., $w_{s}=r_{s}$). The proof of the reliability rs is as follows:

**Proof.**  Suppose that $2^{M}$ pieces of independent evidence are each obtained by Equation (16). It can be known from the basic probability mass function (i.e., Equation (17)) that ${\tilde{m}}_{n,s}=c_{rw,s}m_{n,s}$ for any n⊆Θ and ${\tilde{m}}_{P(\Theta ),s}=c_{rw,s}\left(1-r_{s}\right)$ for *s* = 1,2,…,$2^{M}$.

(17) $$\begin{array}{c} {\tilde{m}}_{n,s}=\left\{\begin{array}{ccc} 0 & & n=\phi \\ c_{rw,s}m_{n,s} & & n\subseteq \Theta ,n\neq \phi \\ c_{rw,s}\left(1-r_{s}\right) & & n=P(\Theta ) \end{array}\right.\\ c_{rw,s}=1/(1+w_{s}-r_{s}) \end{array}$$

From Equation (17), we get $\sum_{n\subseteq \Theta }{\tilde{m}}_{n,s}+{\tilde{m}}_{P(\Theta ),s}=1$. If $w_{s}=r_{s}$, there will be $c_{rw,s}=1$ and ${\tilde{m}}_{n,s}=m_{n,s}$, as well as ${\tilde{m}}_{P(\Theta ),s}=c_{rw,s}\left(1-r_{s}\right)=1-r_{s}$. From the analysis, we can get $r_{s}$ as follows

(18) $$r_{s}=\sum_{n\subseteq \Theta }m_{n,s}=1-m_{P(\Theta ),s}$$

 □

#### C. Evidence Fusion and Ventilation Identification

Through Equation (16), $2^{M}$ pieces of evidence $e_{1},\ldots ,e_{s},\ldots ,e_{2^{M}}$ activated by the input vector $X\left(k\right)=[f_{1}\left(k\right),\ldots ,f_{i}\left(k\right),\ldots ,f_{M}\left(k\right)]$ are obtained. Then, $2^{M}$ pieces of evidence are fused by the ER rule according to Equation (20), and the final result is expressed by Equation (19).

(19) $$O\left(X\left(k\right)\right)=\left\{\left(F_{n},p_{n,e(2^{M})}\right),n=1,2,\ldots ,N\right\}$$

In Equation (19), $p_{n,e(2^{M})}$ indicates the belief degree of the ventilation type $F_{n}$ when the input vector is X(k). In the following model optimization, ${\hat{p}}_{n,k}$ will be used to represent the predicted probability of $F_{n}$ with the input vector X(k), which satisfies ${\hat{p}}_{n,k}=p_{n,e(2^{M})}$.

The recursion formula of the ER rule given in Equation (20) can be used to integrate multiple pieces of evidence $e_{s}$ (*s* = 1,2,…,$2^{M}$).

(20) $$\begin{array}{c} \;\;\;\;\;\;\;\;\;\;\;\;\;\;\;\;\;\;\;\;\;\;\;\;\;\;\;\;\;\;\;\;\;\;\;\;p_{n,e(2^{M})}=\left\{\begin{array}{ccc} 0 & & n=\phi \\ \frac{{\tilde{m}}_{n,e(2^{M})}}{\sum_{D\subseteq \Theta }{\tilde{m}}_{D,e(2^{M})}} & & n\subseteq \Theta ,n\neq \phi \end{array}\right.\\ {\tilde{m}}_{n,e(s)}=\left[\left(1-r_{s}\right)m_{n,e(s-1)}+m_{P(\Theta ),e(s-1)}m_{n,s}\right]+\sum_{B\cap C=n}m_{B,e(s-1)}m_{C,s}\;\;\;\forall n\subseteq \Theta \\ \;\;\;\;\;\;\;\;\;\;\;\;\;\;\;\;\;\;\;\;\;\;\;\;\;\;\;\;\;\;\;\;\;\;\;\;\;\;{\tilde{m}}_{P(\Theta ),e(s)}=\left(1-r_{s}\right)m_{P(\Theta ),e(s-1)} \end{array}$$

According to the result O(X(k)), the ventilation type corresponding to the input vector X(k) can be identified as the type of the maximum degree in O(X(k)).

(21) $$F_{n,k}=\left\{F_{n}|\arg\max p_{n,e(2^{M})}\right\}$$

#### 3.2.3. Intelligent Sea States Identification

This section focuses on the sea states identification that is based on the results of ventilation identification. The criteria for identifying sea state in this article are as follows:

- The sea state is identified as the normal sea state when the propeller is in the non-ventilation region.
- The sea state is identified as the extreme sea state when the propeller is in the partial or full ventilation region (that is, the sea state is normal when the propeller does not produce any ventilation; otherwise, the sea state is extreme).

Assuming that the detection signal of ventilation is ζ, and the type of sea state is denoted by ϕ. When the propeller is not detected to generate ventilation, the signal ζ is set to 0 and the current sea state is recognized as the normal sea state (i.e., ϕ = 0). When the propeller is detected to generate ventilation, the signal ζ is set to 1 and the current sea state is recognized as the extreme sea state (i.e., ϕ = 1). The mathematical relationship is as follows:

(22) $$\phi =\left\{\begin{array}{ccc} 0 & & \zeta =0\\ 1 & & \zeta =1 \end{array}\right.$$

Based on the sea states identification results, different control strategies can be adopted, so that the propulsion system can be well controlled in any sea state.

### 3.3. Optimization of the Ventilation Identification Model Based on GA

The MAKER inference model constructed by the initial parameter set may not accurately capture the complex nonlinear causal relationship between the input feature variable $f_{i}\left(i=1,2,\ldots ,M\right)$ and the output type $F_{n}$. Therefore, these parameters need to be trained using training samples to improve the accuracy of the evaluation model. Specifically, the minimum mean square error (MSE) between the estimated probability (${\hat{p}}_{n,k}$) and the real probability ($p_{n,k}$) of the ventilation type is selected as the objective function to construct the parameter optimization model.

(23) $$\min_{P}\xi \left(P\right)=\frac{1}{K*N}\sum_{k=1}^{K}\sum_{n=1}^{N}{\left(p_{n,k}-{\hat{p}}_{n,k}\right)}^{2}$$

(24) $$s.t.A_{t_{j}-1}^{j}<A_{t_{j}}^{j}<A_{t_{j}+1}^{j},j=1,\ldots ,M;t_{j}=2,\ldots ,T_{j}-1$$

(25) $$0\leq w_{n,t_{i},i}\leq 1,i=1,\ldots ,M;t_{i}=1,\ldots ,T_{i}$$

(26) $$0\leq r_{n,t_{i},i}\leq 1,0\leq \gamma_{B,C,e_{t_{1}}^{1},\ldots ,e_{t_{M}}^{M}}$$

Equations (24)–(26) illustrate the constraints that the fine-tuned parameters need to satisfy. $P=\{A_{t_{j}}^{j},w_{n,t_{i},i},r_{n,t_{i},i},\gamma_{B,C,e_{t_{1}}^{1},\ldots ,e_{t_{M}}^{M}}|j=1,\ldots ,M;t_{j}=1,\ldots ,T_{j};i=1,\ldots ,M;t_{i}=1,\ldots ,T_{i};n=1,\ldots ,N\}$ denotes the parameter set to be optimized, which does not include the boundary value $\min_{k,k\in S_{f_{i}}}\left(f_{i}\left(k\right)\right)$ and $\max_{k,k\in S_{f_{i}}}\left(f_{i}\left(k\right)\right)$. $w_{n,t_{i},i}$ and $r_{n,t_{i},i}$ denote the importance weight and reliability of the evidential elements $e_{t_{i}}^{i}\left(F_{n}\right)$.

**Figure 5:**
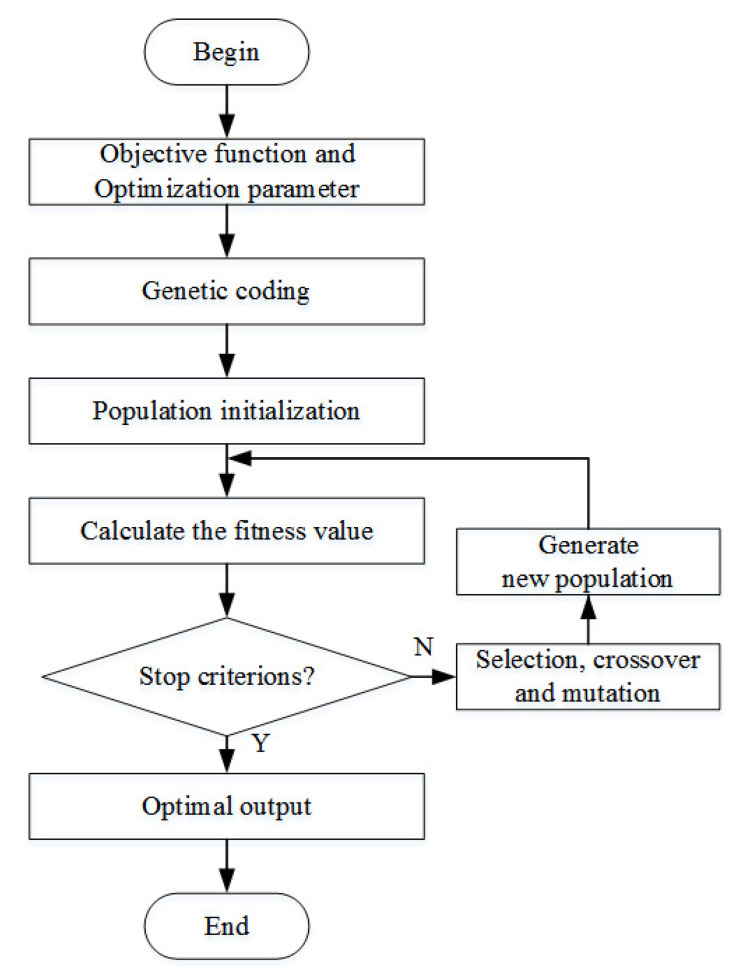
Flow chart of genetic algorithm.

In this paper, GA is used to optimize the parameters of identification model to improve the identification accuracy [26,27]. The flow chart of genetic algorithm is described as in Figure 5 and the specific steps are as follows:

1. The optimization model that is based on MAKER is constructed, and the optimization objective function ξ(P), the optimization parameter set P, and the constraints of the parameters are determined.
2. Genetic coding: the phenotype corresponding to the optimization parameter is mapped to the genotype corresponding to the chromosome or individual.
3. Population initialization: the initial parameter set P is used as an individual of the initial population and (L−1) individuals are randomly generated according to the constraints that the parameters should satisfy, and these L individuals are used as the initial population.
4. Calculate the fitness value: the objective function is used as the fitness function, and the fitness values of *L* individuals are calculated, respectively, and the *L* individuals are ranked according to their corresponding fitness values.
5. Determine optimization stop criterions: when the evolutionary algebra reaches the set value, the GA optimization is stopped and the individual with the greatest fitness is taken as the optimal output. Otherwise, selection, crossover and mutation operations are continued to generate new population, and repeat step 4 to 7.
6. Selection, crossover, and mutation:
  - Selection: taking the survival of the fittest as the criterion, select individuals with higher fitness to continue genetic operations, and eliminate individuals with lower fitness.
  - Crossover and mutation: the main function of crossover and mutation is generate new individuals.
7. Generate the new population: take the new individuals generated by crossover and mutation as the new population, and return to step 4 to recalculate the fitness value.

## 4. Application

### 4.1. Experimental Data

In this section, a simulation model of marine electric propulsion system based on MATLAB/SIMULINK software is built to simulate propeller ventilation and obtain experimental data. The experimental data are divided into training sample set and test sample set, and the intelligent sea states identification model that is based on the MAKER rule is trained and validated.

#### 4.1.1. Simulation of Propeller Ventilation Based on MATLAB/SIMULINK

**Figure 6:**
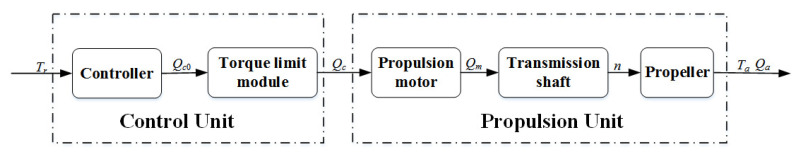
The structure of marine electric propulsion system.

The real marine electric propulsion system is mainly composed of a control unit and propulsion unit, as shown in Figure 6. Specifically, the propulsion unit mainly includes a propulsion motor, a propeller shaft, and a propeller. The control unit mainly includes a propulsion system controller and a torque limiting module. The target thrust $T_{r}$ generated by the propeller acts on the propulsion system controller to generate the target value of the motor torque signal $Q_{c0}$, and then the given value of the propulsion motor torque signal $Q_{c}$ can be obtained via the torque limiting module. Finally, the actual propeller thrust $T_{a}$ and the actual torque $Q_{a}$ are generated through the propulsion unit.

In this paper, the propulsion system parameters and dynamic mathematical models of each module described in reference [28] are used. The simulation model of a marine electric propulsion system is built, as shown in Figure 6, and the propulsion system contains a Wageningen B-series propeller (the number of blades $Z_{w}=4$, the diameter of the propeller $D_{w}$ = 4 m, the pitch ratio $P_{w}/D_{w}=1$) and a permanent magnet synchronous motor (PMSM). The parameters of the propulsion system are shown in Table 5.

#### 4.1.2. Acquiring Input Variables and Sample Data

Based on the simulation model of marine electric propulsion system, the conditions of propeller in non-ventilation region, partial ventilation region, and full ventilation region are simulated, respectively. Additionally, the actual speed n and the actual torque $Q_{m}$ of the propulsion motor in the three regions are collected separately. Subsequently, the sampled parameters $Q_{m}$ and n are converted into parameters that can describe the dynamic performance of the system. The converted feature parameters are used as the input variables of the model (i.e., $f_{1}=n\left(t\right)/n_{bq}$, $f_{2}=n_{r}\left(t\right)-n\left(t\right)$, $f_{3}=\left(n\left(t\right)-n\left(t-1\right)\right)/T_{s}$, $f_{4}=\left(Q_{m}\left(t\right)-Q_{m}\left(t-1\right)\right)/T_{s}))$, and the output types of the model are non-ventilation region $\left(F_{1}\right)$, partial ventilation region $\left(F_{2}\right)$, and full ventilation region $\left(F_{3}\right)$. The sampling period is set to be $T_{s}$ = 0.001 s, and the sample vector $\left[f_{1}\left(k\right),f_{2}\left(k\right),f_{3}\left(k\right),f_{4}\left(k\right),F_{n}\right]$ is obtained by simulation model when the type of ventilation is $F_{n}$. There are 2000 samples for each ventilation type, respectively, and 6000 samples constitute the sample set $S=\{\left[f_{1}\left(k\right),f_{2}\left(k\right),f_{3}\left(k\right),f_{4}\left(k\right),F_{n}\right]|k=1,2,\ldots ,2000;n=1,2,3\}$.

### 4.2. Training the Ventilation Identification Model

The ventilation identification model is trained and tested by the five-fold cross-validation, and the mean value of the five-fold cross-validation is used to estimate of the model precision to improve the precision of the model more. The five-fold cross-validation is to divide the samples in the sample set S into five parts on average, taking four parts of them as the training sample set and the remaining one as the test sample set. In five-fold cross-validation, an identification model can be established by using each training sample set. The following will take the first cross-validation as an example to train the model and illustrate the variation of the training parameters. Where the training sample set $S_{Train}=\left\{\left[f_{1}\left(k\right),f_{2}\left(k\right),f_{3}\left(k\right),f_{4}\left(k\right),F_{n}\right]|k=1,2,\ldots ,1600;n=1,2,3\right\}$ represents 4800 sample data in the sample set S (four parts corresponding to each type of ventilation, i.e., 1600 samples).

The MSE between the estimated probability (${\hat{p}}_{n,k}$) and the real probability ($p_{n,k}$) of the ventilation type is taken as the objective function to construct the parameter optimization model to train the parameters in the set P, as shown in Section 3.3.

(27) $$\begin{array}{c} P=\{A_{t_{j}}^{j},w_{n,t_{i},i},r_{n,t_{i},i},\gamma_{B,C,e_{t_{1}}^{1},\ldots ,e_{t_{M}}^{M}}|j=1,\ldots ,M;\\ \;\;\;\;\;\;\;t_{j}=1,\ldots ,T_{j};i=1,\ldots ,M;t_{i}=1,\ldots ,T_{i};n=1,\ldots ,N\} \end{array}$$

where $M=4,T_{1}=T_{2}=T_{3}=T_{4}=3,N=3$.

In the process of model training and inference, the key parameters are obtained, as follows. In this paper, the initial reference values of $f_{1}$, $f_{2}$, $f_{3}$ and $f_{4}$ are determined by analyzing the input variables in the set $S_{Train}$ statistically, as shown in Table 6. According to the steps A and B in Section 3.2.1, the independence index, IREM and JREM are acquired based on the casting of sample value and the normalization of likelihood function. Subsequently, the predicted probability ${\hat{p}}_{n,k}$ can be obtained from the reasoning process of steps A to C in Section 3.2.2 and the parameters in the set P are optimized by using training samples to improve the accuracy of the evaluation model. It should be noted that the initial parameter $w_{n,t_{i},i}=r_{n,t_{i},i}=\gamma_{B,C,e_{t_{1}}^{1},\ldots ,e_{t_{M}}^{M}}=1$ in the set P. Table 7, Table 8, Table 9 and Table 10 show the reference values of the input $f_{i}$ and the IREM. Table 11 shows the initial JREM of the input vector $\left[f_{1},f_{2}\right]$, and the JREM corresponding to the input vector $\left[f_{1},f_{2},f_{3}\right]$ and $\left[f_{1},f_{2},f_{3},f_{4}\right]$ are also available according to Section 3.2.1.

Table 12, Table 13, Table 14, Table 15 and Table 16 show the optimized IREM and JREM. It can be seen from Table 17, Table 18 and Table 19 that the initial parameters *w*, *r* and γ in the set P have changed. Table 17 and Table 18 list the optimized reliability *r* and importance weight *w* of the evidential elements for the input variable $f_{1}$. Table 19 shows the optimized reliability ratio γ corresponding to the input vector $\left[f_{1},f_{2}\right]$. It can be seen from Table 12, Table 13, Table 14, Table 15 and Table 16 that the input reference value has significantly changed after training, as well as the IREM and JREM. This shows that the model parameters have been adjusted by the training process.

### 4.3. Testing

According to the five-fold cross-validation method, five identification models are constructed, and each of them corresponds to a training sample set and a test sample set. Every model has its own identification accuracy, and the mean value of the five-fold cross-validation is used as as the criterion to evaluate the performance of the ventilation identification model.

#### 4.3.1. The Verification of the Ventilation Identification Model by a Typical Test Samples

The input feature vector $X\left(k\right)=[f_{1}\left(k\right),f_{2}\left(k\right),f_{3}\left(k\right),f_{4}\left(k\right)]=\left[0.6480,0.1866,0.1845,54.5231\right]$ in the test sample set $S_{Test}=\left\{\left[f_{1}\left(k\right),f_{2}\left(k\right),f_{3}\left(k\right),f_{4}\left(k\right),F_{n}\right]|k=1,2,\ldots ,400;n=1,2,3\right\}$ in the first cross-validation is used to describe the inference process of the identification model in detail. 

**Step 1:** acquiring input variables and activated evidence 

According to step A in Section 3.2.1, the similarities of $f_{1}\left(k\right)$ matching $A_{2}^{1}$ and $A_{3}^{1}$ are $\alpha_{1,2}=0.918$ and $\alpha_{1,3}=0.082$, and evidence $e_{2}^{1}$ and $e_{3}^{1}$ are activated. The similarities of $f_{2}\left(k\right)$ matching $A_{2}^{2}$ and $A_{3}^{2}$ are $\alpha_{2,2}=0.3288$ and $\alpha_{2,3}=0.6712$, and evidence $e_{2}^{2}$ and $e_{3}^{2}$ are activated. The similarities of $f_{3}\left(k\right)$ matching $A_{2}^{3}$ and $A_{3}^{3}$ are $\alpha_{3,2}=0.6605$ and $\alpha_{3,3}=0.3395$, and evidence $e_{2}^{3}$ and $e_{3}^{3}$ are activated. The similarities of $f_{4}\left(k\right)$ matching $A_{2}^{4}$ and $A_{3}^{4}$ are $\alpha_{4,2}=0.2468$ and $\alpha_{4,3}=0.7532$, and evidence $e_{2}^{4}$ and $e_{3}^{4}$ are activated. 

**Step 2:** interdependence between pairs of evidence 

According to the step B in Section 3.2.1, the 16 combination of reference evidence are activated by input vector $\left[f_{1}\left(k\right),f_{2}\left(k\right),f_{3}\left(k\right),f_{4}\left(k\right)\right]$, and the interdependence index between multiple pieces of reference evidence can be obtained by Equation (12), as shown in Table 20. 

**Step 3:** acquiring activated evidence 

The MAKER rule in Equation (7) is used to integrate every activated evidences $e_{1},e_{2},\ldots ,e_{16}$. Table 20 shows the combined evidence activated by the kth input feature vector X(k). 

**Step 4:** evidence fusion 

After obtaining 16 pieces of evidence $e_{1},e_{2},\ldots ,e_{16}$, we can use the ER rule in Equation (20) to combine them with weights and reliabilities to yield the fused result. The combined result can be obtained as $O\left(X\left(k\right)\right)=\left\{\left(F_{1},0.9987\right),\left(F_{2},0.0013\right),\left(F_{3},0\right)\right\}$. 

**Step 5:** identification of ventilation and sea states 

Finally, according to the combined result O(X(k)), the ventilation type corresponding to the input vector X(k) is identified as $F_{1}$, which is consistent with the actual ventilation type. Furthermore, the current sea state can be identified as a normal sea state.

#### 4.3.2. Comparison and Analysis of Test Results

The identification of ventilation is the basis of intelligent sea states identification, and the accuracy of the ventilation identification is sufficient for reflecting the accuracy of intelligent sea states identification. In this paper, the accuracy of the ventilation identification results will be used as a criterion to measure the effectiveness of the intelligent sea states identification model. The sample data in the sample set *S* is used to perform a five-fold cross-validation on the identification model, and the mean value of the ventilation identification results in the five identification models is expressed as a confusion matrix to reflect the accuracy of the identification. Table 21 shows the ventilation identification results of the test sample set in the identification model based on the MAKER rule.

It can be seen from Table 21 that the ventilation identification model that is based on the MAKER rule can identify the non-ventilation region ($F_{1}$) and the full ventilation region ($F_{3}$) well, while the identification rate for the partial ventilation region ($F_{2}$) is relatively low, and there is a misidentification. As the propeller immersion ratio h/R increases (decreases) gradually during the process of the propeller entering or exiting the water surface, the ventilation generated by the propeller evolves from full ventilation region $F_{3}$ (non-ventilation region $F_{1}$) to partial ventilation region $F_{2}$, and finally to non-ventilation region $F_{1}$ (full ventilation region $F_{3}$). Whether the propeller enters the water or exits, the partial ventilation state is a transition state. This state is extremely unstable. The input vector collected in this state does not obviously distinguish from the data collected in the other two states, which is also the reason for the misjudgment of $F_{2}$.

The ventilation identification results of the test samples are further analyzed to verify the validity of the identification model built in this paper. Under the same experimental conditions, the ventilation identification performance of the MAKER identification model (after training) proposed in this paper is compared with that of the MAKER identification model (before training), the conventional ER identification model [29,30], the back propagating artificial neutral net (BP-ANN) identification model [31] and support vector machine (SVM) identification model [32].

Table 22, Table 23, Table 24 and Table 25 show the results of the four identification models. The identification result of each ventilation type can be clearly seen from the table. Specifically, as can be seen from Table 22, the overall recognition rates of the MAKER model before and after training are 96.18% and 98.82%, respectively. It can be seen that the problem of insufficient accuracy that is caused by inaccurate initial parameters of the model can be solved after training. Table 23 shows the recognition results of the ER model. The overall recognition rate of the ER model is 96.3%, which is lower than that of the MAKER model (after training). Although the traditional ER identification model can achieve effective identification of the inhalation effect to a certain extent, the ER-based identification model can only be used to fuse mutually independent evidence. Additionally, the interdependence between the evidence is fully considered, which makes up the shortage that ER rule can only combine independent evidence. It can be seen from Table 24 that the overall recognition rate of the BP-ANN model is 98.78%. Compared with the MAKER model (after training), the overall recognition rate of the BP-ANN model is lower, and some samples belonging to F1 are misjudged to F3. This situation does not exist in the MAKER model. Similarly, as can be seen from Table 25, the overall recognition rate of the SVM model is 98.25%, and its overall recognition rate is lower than the MAKER model (after training).

Table 26 shows the results of the four models in the five-fold cross-validation. It can be seen that the overall accuracy of the MAKER model (after training) reaches 98.82%, which is higher than other methods. The overall accuracy of SVM model and BP-ANN model is similar to that of MAKER model (after training), but the accuracy of the MAKER model (after training) is generally higher than that of the other two models in each fold cross-validation, which shows that the MAKER model (after training) has universal applicability.

When considering that the partial ventilation state is extremely unstable, and is prone to misjudgment, Table 26 lists the average recognition rate of partial ventilation ($F_{2}$) of the four recognition methods. It can be seen that the average recognition rate of the MAKER model (after training) for $F_{2}$ is 96.5%, which is higher than the traditional ER model and the BP-ANN model. Besides, the BP-ANN method is a “black box” system. The physical meaning of the system parameters in the BP-ANN model is unclear, not readily understood, and unadjustable, while the physical meaning of the parameters in the MAKER method is clear, readily understood, and adjustable. The average recognition rate of $F_{2}$ recognized by the SVM model is 97%, which is slightly higher than the average recognition rate of $F_{2}$ given by the MAKER model (after training). However, the recognition rate of the MAKER model (after training) under each cross-validation is generally higher than that of the SVM recognition model. Simultaneously, SVM has limitations. Specifically, SVM is a binary classification algorithm, which is difficult to solve the multi-classification problem, and can only use the “OneVsone” and “OneVsRest” strategies, while a multi-input and multi-output classification system can be directly constructed based on MAKER rule. Based on the likelihood function normalization, the MAKER rule model can avoid small samples to be submerged in large samples in ventilation identification, which ensures the small probability events, even the abnormal samples rarely occurring to be recognized effectively. According to the sampling method presented in Section 4.1, a total samples of 520 are collected to construct $S_{1}$, in which there are 400 samples for $F_{1}$, 100 samples for $F_{2}$, and 20 samples for $F_{3}$. The test sample set contains 104 samples, in which there are 80 samples for $F_{1}$, 20 samples for $F_{2}$ and 4 samples for $F_{3}$. Table 27 shows the ventilation identification results of the test sample set in $S_{1}$ based on the MAKER rule and SVM. Table 28 shows the results of 5-fold cross-validation of MAKER and SVM for the test samples in $S_{1}$. It can be seen from Table 27 and Table 28 that the recognition rate of test sample set by the MAKER model is 99.42%, which is higher than the recognition rate of SVM. It can be concluded that MAKER can better handle small probability samples and unbalanced sample sets.

From the analysis of the results of the five-fold cross-validation, it can be seen that the intelligent sea states identification model that is based on the MAKER rule proposed in this paper performs better than other models on ventilation identification, and finally identify the sea states accurately. Based on the sea states identification result, we could adopt the appropriate control strategy, so that the propulsion system can be well controlled in any sea state.

## 5. Conclusions

This paper proposes an intelligent sea states identification model based on the MAKER rule to identify the current sea state effectively and choose the suitable control strategy for propulsion system. An identification model of ventilation based on the MAKER rule is established to identify the ventilation type first, and then the sea state is identified based on the correspondence between the type of propeller ventilation and sea states. The main work of this article is as follows.

In this paper, an intelligent sea states identification model is established that is based on the correspondence between the type of propeller ventilation and sea states. The independence index, IREM and JREM are acquired based on the casting of sample value and the normalization of likelihood function. Under the premise of fully considering the independence between the input features, the reliability and importance weight of the evidence, the MAKER rule is used to estimate the type of the ventilation, and the sea state is identified according to the recognition result of the ventilation. In the process of evidence fusion using the MAKER rule, this paper proposes a method for obtaining the reliability factor of evidence that is based on the basic probability mass function. Finally, a simulation model of a marine electric propulsion system that is based on MATLAB/SIMULINK software is built to obtain experimental data, and GA is used to train the ventilation identification model that is based on the MAKER rule, and the validity of the model is verified. Furthermore, by comparing and analyzing the results of the proposed method and other typical methods in the five-fold cross-validation, it can be seen that the sea states identification model based on MAKER rule proposed in this paper can more accurately identify the propeller ventilation, and finally realize intelligent identification of sea states.

The intelligent sea states identification model that is proposed in this paper can realize the effective identification of sea states. According to the change of sea states, the ship can switch the control strategy of propulsion system in time in order to ensure the safe navigation. Specifically, the sea state is identified as the normal sea state and a speed control strategy, a torque control strategy, or a power control strategy can be used to realize the control of propulsion system when the propeller is in the non-ventilation region. Otherwise, the sea state is identified as the extreme sea state and the anti-spin control strategy can be adopted to reduce the impact of ventilation on the ship when the propeller is in the partial or full ventilation region. The work on sea states identification has a positive contribution to maritime safety.

The intelligent identification model that is based on the MAKER rule is a data-driven reasoning method, which does not need to make any assumptions about the relationships between input variables and output parameters, reducing dependence on expert domain knowledge, and the parameters of the model are clear in physical meaning, readily understood, and adjustable. Additionally, the identification model based on MAKER rule can be expanded to a general classifier. It is not only suitable for the ventilation recognition, but also suitable for similar classification or evaluation problems in which there are insufficient data samples and the dependence among the input attributes should be considered. Through the casting of sample value and the normalization of likelihood function, this method can also realize the parameter estimation by using incomplete data samples, which is of great significance for the study of incomplete data sample classification. However, a lot of work should be further conducted in order to improve the model performance and navigation safety. The future work directions are as follows: (1) validate the proposed identification method based on real-world datasets of actual engineering systems; and, (2) propose the control strategy of electric propulsion system, and refine the parameter training process. Subsequently, the ventilation identification and control strategy are combined to realize the dynamic identification of ventilation and the switch of different control strategies.

## Figures and Tables

**Table 1:** Casting results of sample pairs $\left(f_{i},F_{n}\right)$ depend on the reference values.

F	$f_{i}$					Total
	$A_{1}^{i}$	⋯	$A_{t_{i}}^{i}$	⋯	$A_{T_{i}}^{i}$	
$F_{1}$	$a_{1,1}$	⋯	$a_{1,t_{i}}$	⋯	$a_{1,T_{i}}$	$\delta_{1}$
⋮	⋮	⋮	⋮	⋮	⋮	⋮
$F_{n}$	$a_{n,1}$	⋯	$a_{n,t_{i}}$	⋯	$a_{n,T_{i}}$	$\delta_{n}$
⋮	⋮	⋮	⋮	⋮	⋮	⋮
$F_{N}$	$a_{N,1}$	⋯	$a_{N,t_{i}}$	⋯	$a_{N,T_{i}}$	$\delta_{N}$
Total	$\eta_{1}$	⋯	$\eta_{t_{i}}$	⋯	$\eta_{T_{i}}$	*K*

**Table 2:** The initial reference evidence matrix (IREM) of the input $f_{i}$.

F	$e_{1}^{i}$	⋯	$e_{t_{i}}^{i}$	⋯	$e_{T_{i}}^{i}$
	$A_{1}^{i}$	⋯	$A_{t_{i}}^{i}$	⋯	$A_{T_{i}}^{i}$
$F_{1}$	$\beta_{1,1}^{i}$	⋯	$\beta_{1,t_{i}}^{i}$	⋯	$\beta_{1,T_{i}}^{i}$
⋮	⋮	⋮	⋮	⋮	⋮
$F_{n}$	$\beta_{n,1}^{i}$	⋯	$\beta_{n,t_{i}}^{i}$	⋯	$\beta_{n,T_{i}}^{i}$
⋮	⋮	⋮	⋮	⋮	⋮
$F_{N}$	$\beta_{N,1}^{i}$	⋯	$\beta_{N,t_{i}}^{i}$	⋯	$\beta_{N,T_{i}}^{i}$

**Table 3:** The joint casting result of sample pairs $\left(\left[f_{1},f_{2}\right],F_{n}\right)$.

F	$A_{1}^{1}$					⋯	$A_{T_{1}}^{1}$					Total
	$A_{1}^{2}$	⋯	$A_{t_{2}}^{2}$	⋯	$A_{T_{2}}^{2}$	⋯	$A_{1}^{2}$	⋯	$A_{t_{2}}^{2}$	⋯	$A_{T_{2}}^{2}$	
$F_{1}$	$a_{1,1}^{1}$	⋯	$a_{1,t_{2}}^{1}$	⋯	$a_{1,T_{2}}^{1}$	⋯	$a_{T_{1},1}^{1}$	⋯	$a_{T_{1},t_{2}}^{1}$	⋯	$a_{T_{1},T_{2}}^{1}$	$\delta_{1}$
⋮	⋮	⋮	⋮	⋮	⋮	⋮	⋮	⋮	⋮	⋮	⋮	⋮
$F_{n}$	$a_{1,1}^{n}$	⋯	$a_{1,t_{2}}^{n}$	⋯	$a_{1,T_{2}}^{n}$	⋯	$a_{T_{1},1}^{n}$	⋯	$a_{T_{1},t_{2}}^{n}$	⋯	$a_{T_{1},T_{2}}^{n}$	$\delta_{n}$
⋮	⋮	⋮	⋮	⋮	⋮	⋮	⋮	⋮	⋮	⋮	⋮	⋮
$F_{N}$	$a_{1,1}^{N}$	⋯	$a_{1,t_{2}}^{N}$	⋯	$a_{1,T_{2}}^{N}$	⋯	$a_{T_{1},1}^{N}$	⋯	$a_{T_{1},t_{2}}^{N}$	⋯	$a_{T_{1},T_{2}}^{N}$	$\delta_{N}$
Total	$\eta_{1,1}$	⋯	$\eta_{1,t_{2}}$	⋯	$\eta_{1,T_{2}}$	⋯	$\eta_{T_{1},1}$	⋯	$\eta_{T_{1},t_{2}}$	⋯	$\eta_{T_{1},T_{2}}$	*K*

**Table 4:** The joint reference evidence matrix (JREM) of the input $\left[f_{1},f_{2}\right]$.

F	$A_{1}^{1}$					⋯	$A_{T_{1}}^{1}$				
	$A_{1}^{2}$	⋯	$A_{t_{2}}^{2}$	⋯	$A_{T_{2}}^{2}$	⋯	$A_{1}^{2}$	⋯	$A_{t_{2}}^{2}$	⋯	$A_{T_{2}}^{2}$
	$e_{1,1}^{1,2}$	⋯	$e_{1,t_{2}}^{1,2}$	⋯	$e_{1,T_{2}}^{1,2}$	⋯	$e_{T_{1},1}^{1,2}$	⋯	$e_{T_{1},t_{2}}^{1,2}$	⋯	$e_{T_{1},T_{2}}^{1,2}$
$F_{1}$	$\beta_{1,1,1}^{1,2}$	⋯	$\beta_{1,1,t_{2}}^{1,2}$	⋯	$\beta_{1,1,T_{2}}^{1,2}$	⋯	$\beta_{1,T_{1},1}^{1,2}$	⋯	$\beta_{1,T_{1},t_{2}}^{1,2}$	⋯	$\beta_{1,T_{1},T_{2}}^{1,2}$
⋮	⋮	⋮	⋮	⋮	⋮	⋮	⋮	⋮	⋮	⋮	⋮
$F_{n}$	$\beta_{n,1,1}^{1,2}$	⋯	$\beta_{n,1,t_{2}}^{1,2}$	⋯	$\beta_{n,1,T_{2}}^{1,2}$	⋯	$\beta_{n,T_{1},1}^{1,2}$	⋯	$\beta_{n,T_{1},t_{2}}^{1,2}$	⋯	$\beta_{n,T_{1},T_{2}}^{1,2}$
⋮	⋮	⋮	⋮	⋮	⋮	⋮	⋮	⋮	⋮	⋮	⋮
$F_{N}$	$\beta_{N,1,1}^{1,2}$	⋯	$\beta_{N,1,t_{2}}^{1,2}$	⋯	$\beta_{N,1,T_{2}}^{1,2}$	⋯	$\beta_{N,T_{1},1}^{1,2}$	⋯	$\beta_{N,T_{1},t_{2}}^{1,2}$	⋯	$\beta_{N,T_{1},T_{2}}^{1,2}$

**Table 5:** Parameters of marine electric propulsion system.

Parameter	Value	Parameter	Value
Rated motor torque $Q_{N}$	78 kNm	Nominal thrust coefficient $K_{T}$	0.445
Rated motor power $P_{N}$	4000 kW	Nominal torque coefficient $K_{Q}$	0.0666
Rated motor speed $n_{N}$	8.2 rps	Rotational inertia $I_{s}$	25,000 kgm${}^{2}$
Maximum thrust of propeller $T_{bq}$	490 kN	Friction coefficient $K_{w}$	350 Nms
Maximum power of propeller $P_{bq}$	3800 kW	Maximum speed of propeller $n_{bq}$	2.05 rps
Gearbox reduction ratio $k_{g}$	4	Motor time constant $T_{m}$	0.001 s

**Table 6:** Initial reference values of inputs.

Input	Reference Values		
Input 1$\left(f_{1}\right)$	0.5	0.6	1.1
Input 2$\left(f_{2}\right)$	−0.7	−0.05	0.31
Input 3$\left(f_{3}\right)$	−0.6	0.03	0.85
Input 4$\left(f_{4}\right)$	−127	−16.5	78

**Table 7:** IREM of the input $f_{1}$.

F	$A_{1}^{1}$	$A_{2}^{1}$	$A_{3}^{1}$
	0.5	0.6	1.1
$F_{1}$	0	0.4363	0.17
$F_{2}$	0	0.3438	0.3187
$F_{3}$	1	0.2199	0.5113

**Table 8:** IREM of the input $f_{2}$.

F	$A_{1}^{2}$	$A_{2}^{2}$	$A_{3}^{2}$
	−0.7	−0.05	0.31
$F_{1}$	0.0191	0.3164	0.7127
$F_{2}$	0.1080	0.4996	0
$F_{3}$	0.8729	0.1840	0.2873

**Table 9:** IREM of the input $f_{3}$.

F	$A_{1}^{3}$	$A_{2}^{3}$	$A_{3}^{3}$
	−0.6	0.03	0.85
$F_{1}$	0.1019	0.4165	0.1103
$F_{2}$	0	0.3906	0.3839
$F_{3}$	0.8981	0.1929	0.5058

**Table 10:** IREM of the input $f_{4}$.

F	$A_{1}^{4}$	$A_{2}^{4}$	$A_{3}^{4}$
	−127	−16.5	78
$F_{1}$	0	0.2863	0.6291
$F_{2}$	0	0.5070	0.1617
$F_{3}$	1	0.2067	0.2092

**Table 11:** The joint reference evidence matrix (JREM) of the input vector $\left[f_{1},f_{2}\right]$.

F	$A_{1}^{1}$			$A_{2}^{1}$			$A_{3}^{1}$		
	$A_{1}^{2}$	$A_{2}^{2}$	$A_{3}^{2}$	$A_{1}^{2}$	$A_{2}^{2}$	$A_{3}^{2}$	$A_{1}^{2}$	$A_{2}^{2}$	$A_{3}^{2}$
	[0.5,−0.7]	[0.5,−0.05]	[0.5,0.31]	[0.6,−0.7]	[0.6,−0.05]	[0.6,0.31]	[1.1,−0.7]	[1.1,−0.05]	[1.1,0.31]
$F_{1}$	0	0	0	0.0494	0.3593	0.7169	0.0087	0.2444	0.8463
$F_{2}$	0	0	0	0.2653	0.5057	0	0.0544	0.4893	0
$F_{3}$	1	1	1	0.6853	0.1350	0.2831	0.9369	0.2663	0.1537

**Table 12:** The optimized IREM of the input $f_{1}$.

F	$A_{1}^{1}$	$A_{2}^{1}$	$A_{3}^{1}$
	0.5	0.6076	1.1
$F_{1}$	0	0.4390	0.1629
$F_{2}$	0	0.3460	0.3175
$F_{3}$	1	0.2150	0.5196

**Table 13:** The optimized IREM of the input $f_{2}$.

F	$A_{1}^{2}$	$A_{2}^{2}$	$A_{3}^{2}$
	−0.7	−0.0653	0.31
$F_{1}$	0	0.3137	0.7123
$F_{2}$	0.0733	0.5032	0
$F_{3}$	0.9267	0.1831	0.2877

**Table 14:** The optimized IREM of the input $f_{3}$.

F	$A_{1}^{3}$	$A_{2}^{3}$	$A_{3}^{3}$
	−0.6	−0.1576	0.85
$F_{1}$	0	0.4168	0.2617
$F_{2}$	0	0.3600	0.4119
$F_{3}$	1	0.2232	0.3264

**Table 15:** The optimized IREM of the input $f_{4}$.

F	$A_{1}^{4}$	$A_{2}^{4}$	$A_{3}^{4}$
	−127	−17.1408	78
$F_{1}$	0	0.2858	0.6257
$F_{2}$	0	0.5059	0.1670
$F_{3}$	1	0.2083	0.2073

**Table 16:** The optimized reference evidence matrix (JREM) of the input vector $\left[f_{1},f_{2}\right]$.

F	$A_{1}^{1}$			$A_{2}^{1}$			$A_{3}^{1}$		
	$A_{1}^{2}$	$A_{2}^{2}$	$A_{3}^{2}$	$A_{1}^{2}$	$A_{2}^{2}$	$A_{3}^{2}$	$A_{1}^{2}$	$A_{2}^{2}$	$A_{3}^{2}$
	[0.5,−0.7]	[0.5,−0.0653]	[0.5,0.31]	[0.6067,−0.7]	[0.6067,−0.0653]	[0.6067,0.31]	[1.1,−0.7]	[1.1,−0.0653]	[1.1,0.31]
$F_{1}$	0	0	0	0	0.3559	0.7322	0	0.2405	0.7910
$F_{2}$	0	0	0	0.1989	0.5108	0	0.0355	0.4902	0
$F_{3}$	1	1	1	0.8011	0.1333	0.2678	0.9645	0.2693	0.2090

**Table 17:** The optimized importance weight *w* of the input $f_{1}$.

F	$e_{1}^{1}$	$e_{2}^{1}$	$e_{3}^{1}$
$F_{1}$	0.9306	0.8205	0.8440
$F_{2}$	0.7951	0.9071	0.8209
$F_{3}$	0.8403	0.9109	0.8474

**Table 18:** The optimized reliability *r* of the input $f_{1}$.

F	$e_{1}^{2}$	$e_{2}^{2}$	$e_{3}^{2}$
$F_{1}$	0.8063	0.8782	0.9205
$F_{2}$	0.9566	0.8515	0.9038
$F_{3}$	0.9322	0.8268	0.9757

**Table 19:** The optimized reliability ratio of of the input vector $\left[f_{1},f_{2}\right]$.

F	$e_{1}^{1}$			$e_{2}^{1}$			$e_{3}^{1}$		
	$e_{1}^{2}$	$e_{2}^{2}$	$e_{3}^{2}$	$e_{1}^{2}$	$e_{2}^{2}$	$e_{3}^{2}$	$e_{1}^{2}$	$e_{2}^{2}$	$e_{3}^{2}$
$F_{1}$	0.8063	0.8782	0.9205	0.9731	0.8949	0.8490	0.9399	0.8994	0.8883
$F_{1}$	0.9566	0.8515	0.9038	0.7019	0.8439	0.8817	0.8524	0.8840	0.7853
$F_{1}$	0.9322	0.8268	0.9757	0.9450	0.9051	0.9345	0.9755	0.9135	0.8944

**Table 20:** The fusion process parameters of the input vector $\left[f_{1},f_{2}\right]$.

InputVector	CombinationofEvidence	InterdependenceIndex		
		$\left[f_{1},f_{2}\right]$	$\left[f_{1},f_{2},f_{3}\right]$	$\left[f_{1},f_{2},f_{3},f_{4}\right]$
$\begin{array}{c} X(k)=\\ {}[0.6480,\\ 0.1866,\\ 0.1845,\\ 54.5231] \end{array}$	$\left[e_{2}^{1},e_{2}^{2},e_{2}^{3},e_{2}^{4}\right]$	[2.58,2.93,3.38]	[7.58,8.52,3.53]	[23.44,18.81,10.45]
	$\left[e_{2}^{1},e_{2}^{2},e_{2}^{3},e_{3}^{4}\right]$	[2.58,2.93,3.38]	[7.58,8.52,3.53]	[17.31,32.45,21.25]
	$\left[e_{2}^{1},e_{2}^{2},e_{3}^{3},e_{2}^{4}\right]$	[2.58,2.93,3.38]	[7.23,8.72,8.9]	[19.75,19.05,39.42]
	$\left[e_{2}^{1},e_{2}^{2},e_{3}^{3},e_{3}^{4}\right]$	[2.58,2.93,3.38]	[7.23,8.72,8.9]	[24.18,36.6,6.21]
	$\left[e_{2}^{1},e_{3}^{2},e_{2}^{3},e_{2}^{4}\right]$	[2.34,0,4.33]	[5.64,0,19.23]	[24.2,0,34.37]
	$\left[e_{2}^{1},e_{3}^{2},e_{2}^{3},e_{3}^{4}\right]$	[2.34,0,4.33]	[5.64,0,19.23]	[8.70,0,101.41]
	$\left[e_{2}^{1},e_{3}^{2},e_{3}^{3},e_{2}^{4}\right]$	[2.34,0,4.33]	[9.78,0,9.89]	[41.32,0,7.99]
	$\left[e_{2}^{1},e_{3}^{2},e_{3}^{3},e_{3}^{4}\right]$	[2.34,0,4.33]	[9.78,0,9.89]	[15.1,0,54.17]
	$\left[e_{3}^{1},e_{2}^{2},e_{2}^{3},e_{2}^{4}\right]$	[4.70,3.07,2.83]	[15.71,9.85,4.65]	[56.28,21.27,8.66]
	$\left[e_{3}^{1},e_{2}^{2},e_{2}^{3},e_{3}^{4}\right]$	[4.70,3.07,2.83]	[15.71,9.85,4.65]	[33.38,56.44,2.96]
	$\left[e_{3}^{1},e_{2}^{2},e_{3}^{3},e_{2}^{4}\right]$	[4.70,3.07,2.83]	[10.96,8.57,9.32]	[38.60,18.87,34.67]
	$\left[e_{3}^{1},e_{2}^{2},e_{3}^{3},e_{3}^{4}\right]$	[4.70,3.07,2.83]	[10.96,8.57,9.32]	[36.17,63.46,0]
	$\left[e_{3}^{1},e_{3}^{2},e_{2}^{3},e_{2}^{4}\right]$	[6.82,0,1.4]	[19.19,0,2.16]	[58.3,0,27.95]
	$\left[e_{3}^{1},e_{3}^{2},e_{2}^{3},e_{3}^{4}\right]$	[6.82,0,1.4]	[19.19,0,2.16]	[31.86,0,5.19]
	$\left[e_{3}^{1},e_{3}^{2},e_{3}^{3},e_{2}^{4}\right]$	[6.82,0,1.4]	[32.92,0,0]	[115.2,0,0]
	$\left[e_{3}^{1},e_{2}^{2},e_{3}^{3},e_{3}^{4}\right]$	[6.82,0,1.4]	[32.92,0,0]	[52.61,0,0]

**Table 21:** Average confusion matrix of identification results.

		PredictedType			Total	Average Accuracy (%)	Overall Accuracy(%)
		$F_{1}$	$F_{2}$	$F_{3}$			
**Actual type**	$F_{1}$	399.8	0.2	0	400	99.95	98.82
	$F_{2}$	9	386	5	400	96.5	
	$F_{3}$	0	0	400	400	100	

**Table 22:** Average confusion matrix of MAKER identification model (before training).

		PredictedType			Total	Overall Accuracy (%)
		$F_{1}$	$F_{2}$	$F_{3}$		
**Actual type**	$F_{1}$	388.2	0.8	0	400	96.18
	$F_{2}$	2	377	21	400	
	$F_{3}$	0	11	389	400	

**Table 23:** Average confusion matrix of evidential reasoning (ER) identification model.

		PredictedType			Total	Overall Accuracy (%)
		$F_{1}$	$F_{2}$	$F_{3}$		
**Actual type**	$F_{1}$	387.4	12.6	0	400	96.3
	$F_{2}$	5	379	16	400	
	$F_{3}$	0	10.8	389.2	400	

**Table 24:** Average confusion matrix of back propagating artificial neutral net (BP-ANN) identification model.

		PredictedType			Total	Overall Accuracy (%)
		$F_{1}$	$F_{2}$	$F_{3}$		
**Actual type**	$F_{1}$	399.6	0	0.4	400	98.78
	$F_{2}$	6	385.8	8.2	400	
	$F_{3}$	0	0	400	400	

**Table 25:** Average confusion matrix of support vector machine (SVM) identification model.

		PredictedType			Total	Overall Accuracy (%)
		$F_{1}$	$F_{2}$	$F_{3}$		
**Actual type**	$F_{1}$	393	1	6	400	98.25
	$F_{2}$	3	388	9	400	
	$F_{3}$	0	2	398	400	

**Table 26:** Results of 5-fold cross-validation of four identification models.

IdentificationModel	Accuracyof5-FoldCross-Validation(%)					Accuracy ($F_{2}$,%)	Overall Accuracy(%)
	1-Fold	2-Fold	3-Fold	4-Fold	5-Fold		
MAKER(after training)	96.83	100	100	97.25	100	96.5	98.82
MAKER(before training)	95.25	96	95.75	96.08	97.83	94.25	96.18
ER	95.08	97.75	96.33	94.67	97.63	94.75	96.3
BP-ANN	94.5	99.83	99.67	99.92	100	96.25	98.78
SVM	96.75	100	98.25	96.25	100	97	98.25

**Table 27:** Average confusion matrix of identification results for MAKER/SVM.

		PredictedType			Total	Overall Accuracy (%)
		$F_{1}$(MAKER/SVM)	$F_{2}$(MAKER/SVM)	$F_{3}$(MAKER/SVM)		
**Actual type**	$F_{1}$	80/80	0/0	0/0	80	99.42/97.11
	$F_{2}$	0/0	20/20	0/0	20	
	$F_{3}$	0.6/3	0/0	3.4/1	4	

**Table 28:** Results of five-fold cross-validation of MAKER and SVM for unbalanced test sample set.

IdentificationModel	Accuracyof5-FoldCross-Validation(%)					Overall Accuracy (%)
	1-Fold	2-Fold	3-Fold	4-Fold	5-Fold	
MAKER	100	100	98.08	99.04	100	99.42
SVM	97.12	98.08	96.15	97.12	97.12	97.11
